# Supplementary material for: Cell geometry and the cytoskeleton impact the nucleo-cytoplasmic localisation of the SMYD3 methyltransferase
Source: Sci Rep. 2020 Nov 26;10:20598. doi: 10.1038/s41598-020-75833-9 (PMC7691988; doi:10.1038/s41598-020-75833-9)
Supplement: Supplementary file 1 — Supplementary Information [file 41598_2020_75833_MOESM1_ESM.pdf]

Supplementary Information

Cell geometry and the cytoskeleton impact the nucleo-cytoplasmic localisation of the SMYD3 methyltransferase

David Pereira[1, 2], Alain Richert[1], Souhila Medjkane[2], Sylvie Hénon[1*], Jonathan B Weitzman[2*]

[1] Université de Paris, CNRS, Matière et Systèmes Complexes (MSC), UMR 7057, F-75006 Paris, France

[2] Université de Paris, CNRS, Epigenetics and Cell Fate (EDC), UMR 7216, F-75006 Paris, France

[*] Corresponding authors: jonathan.weitzman@u-paris.fr , sylvie.henon@u-paris.fr

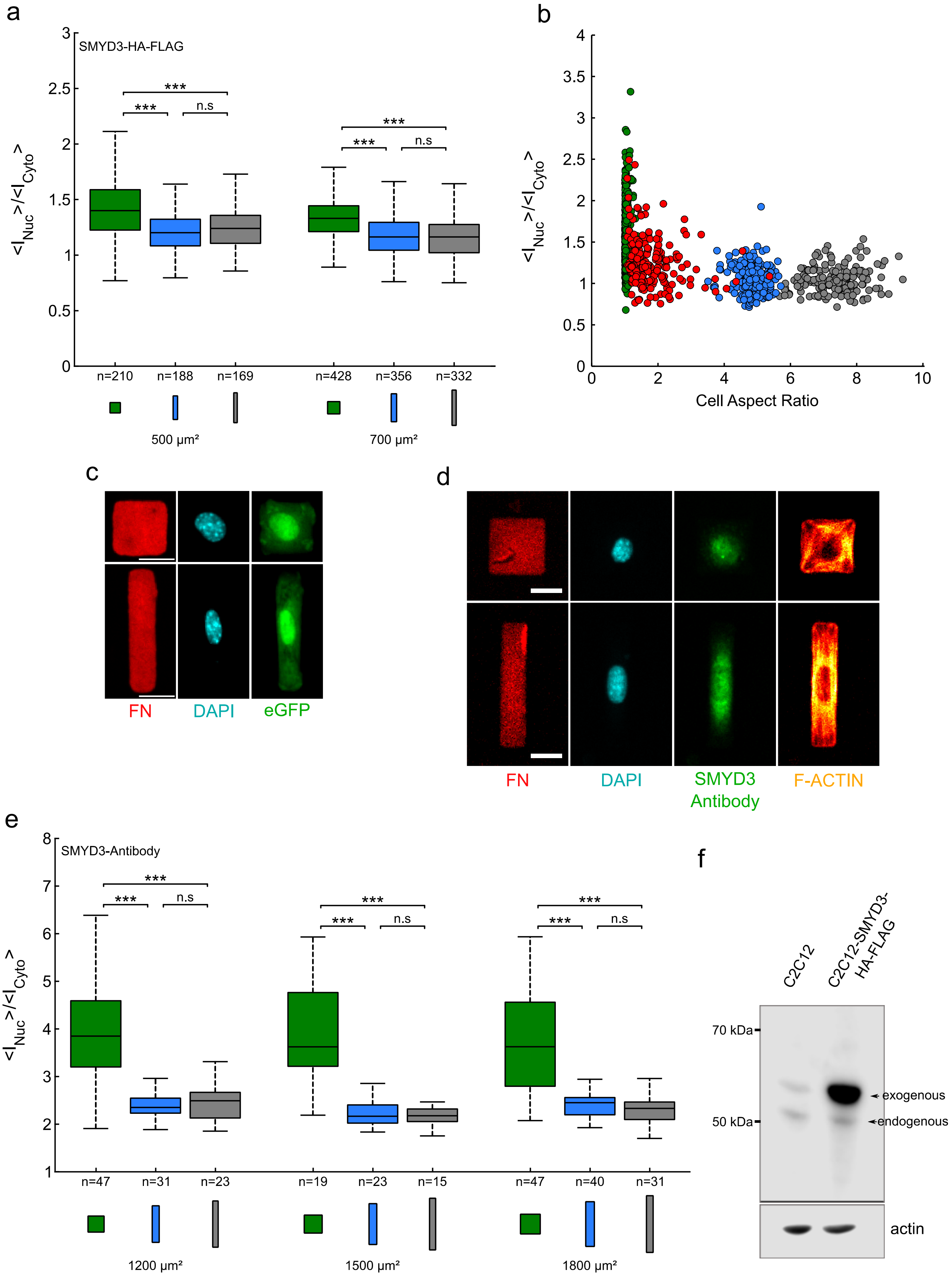
a
SMYD3-HA-FLAG
$\langle I_{Nuc} \rangle / \langle I_{Cyto} \rangle$
***
***
n.s
n=210
n=188
n=169
n=428
n=356
n=332
500 µm²
700 µm²
b
$\langle I_{Nuc} \rangle / \langle I_{Cyto} \rangle$
Cell Aspect Ratio
c
FN
DAPI
eGFP
d
FN
DAPI
SMYD3
Antibody
F-ACTIN
e
SMYD3-Antibody
$\langle I_{Nuc} \rangle / \langle I_{Cyto} \rangle$
n=47
n=31
n=23
n=19
n=23
n=15
n=47
n=40
n=31
1200 µm²
1500 µm²
1800 µm²
f
C2C12
C2C12-SMYD3-HA-FLAG
70 kDa
50 kDa
exogenous
endogenous
actin

Figure S1: **Cell geometry on fibronectin micropatterns regulates exogenous and endogenous SMYD3 distribution.**

a) Quantification of the nuclear:cytoplasmic (<I_{Nuc}>/<I_{Cyto}>) distribution ratio for exogenous SMYD3-HA-Flag on small pattern areas (500 and 700 µm²) and geometries: squares (1:1 aspect ratio, green), rectangles (1:5 aspect ratio, blue) and elongated rectangles (1:8 aspect ratio, grey). The median value of the ratio is about 15% higher on squares than on rectangle patterns. A Kruskal-Wallis test was performed for conditions with identical areas.

b) Representation of the nuclear:cytoplasmic ratio of SMYD3-HA-Flag as a function of cell aspect ratio for cells spread on non-patterned substrates (red, n=202), squares (1:1 aspect ratio, green, n=395), rectangles (1:5 aspect ratio, blue, n=252) and elongated rectangles (1:8 aspect ratio, grey, n=163).

c) Micrographs show the fibronectin (FN) patterning, the nuclear DNA staining (DAPI), and localization of control eGFP in C2C12 cells plated on square or rectangle patterns (1200 µm²). See Figure 1e for quantification data.

d) Non-transfected C2C12 cells were plated on different fibronectin micropatterns with the same area (1200 µm²), but different aspect ratios; square (1:1, upper panels) or rectangle (1:5, lower panels). The micrographs show the fibronectin (FN) patterning, the nuclear DNA staining (DAPI), endogenous SMYD3 protein detected with a specific antibody (SMYD3) and F-actin.

e) Quantification of the nuclear:cytoplasmic (<I_{Nuc}>/<I_{Cyto}>) distribution ratio for endogenous SMYD3 protein over a range of pattern areas (1200-1800 µm²) and geometries: squares (1:1 aspect ratio, green), rectangles (1:5 aspect ratio, blue) and elongated rectangles (1:8 aspect ratio, grey). The median value of the ratio is 48 to 67% higher on squares than on rectangle patterns. A Kruskal-Wallis test was performed for conditions with identical areas.

f) Western blot analysis of exogenous SMYD3-HA-FLAG levels compared to endogenous SMYD3 in control C2C12 cells.

n = number of individual cells measured. *** p<0.001, n.s; = not statistically significant.

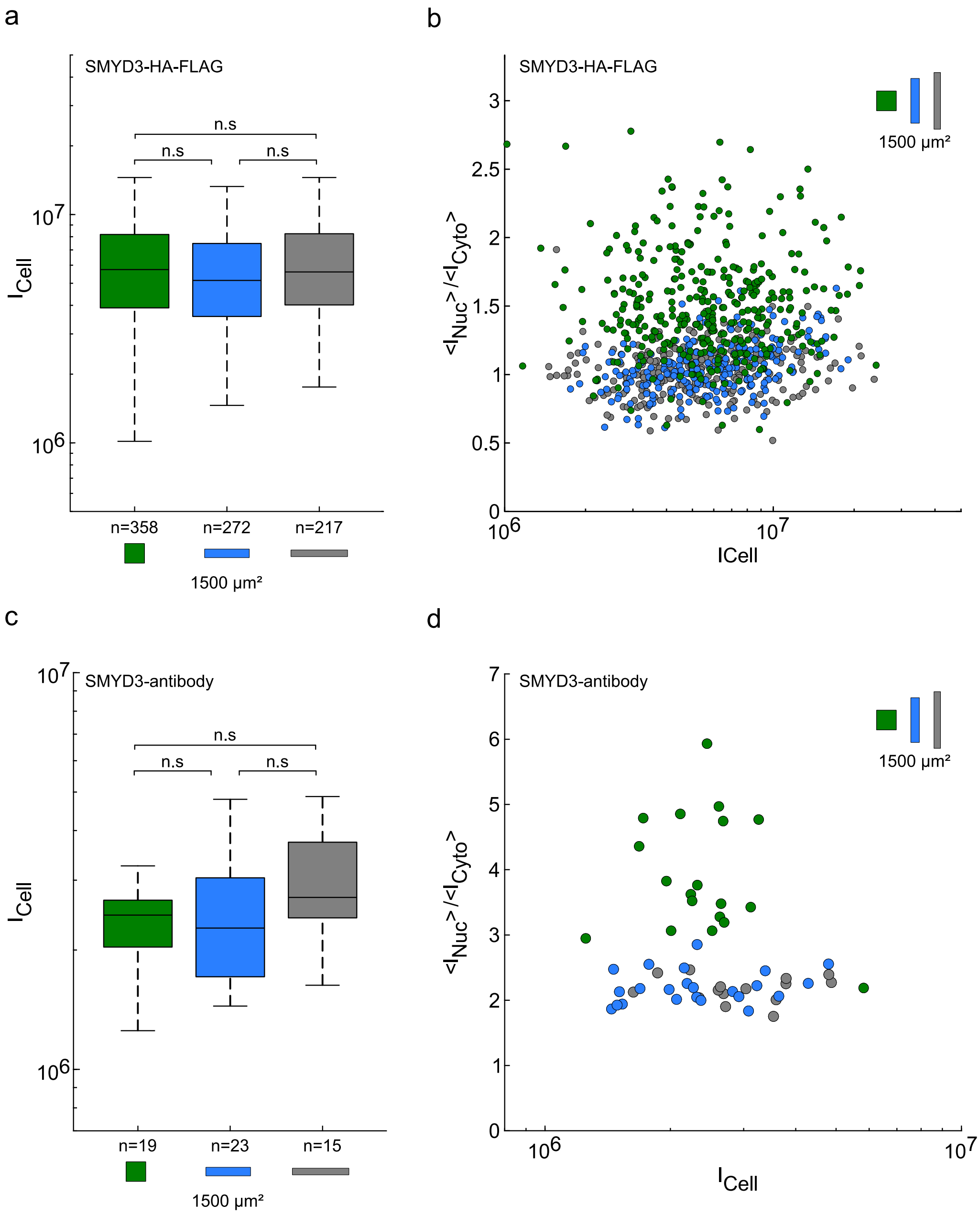

Figure S2: **Cell geometry does not affect the total intensity of SMYD3**

a) Quantification of the total cell intensity of exogenous SMYD3-HA-Flag on pattern area (1500 µm²) of different geometries: squares (1:1 aspect ratio, green), rectangles (1:5 aspect ratio, blue) and elongated rectangles (1:8 aspect ratio, grey). n = number of individual cells measured. An ANOVA test was performed for conditions with identical areas (1500µm²). n.s; = not statistically significant

b) Quantification of the nuclear:cytoplasmic ($<I_{Nuc}>/<I_{Cyto}>$) distribution ratio for exogenous SMYD3-HA-Flag on pattern area (1500 µm²) compare to the total intensity for each cell.

c) Quantification of the total cell intensity of endogenous SMYD3 on pattern area (1500 µm²) of different geometries: squares (1:1 aspect ratio, green), rectangles (1:5 aspect ratio, blue) and elongated rectangles (1:8 aspect ratio, grey). n = number of individual cells measured.

d) Quantification of the nuclear:cytoplasmic ($<I_{Nuc}>/<I_{Cyto}>$) distribution ratio for endogenous SMYD3 on pattern area (1500 µm²) compare to the total intensity for each cell. n = number of individual cells measured. An ANOVA test was performed for conditions with identical areas (1500µm²). n.s; = not statistically significant

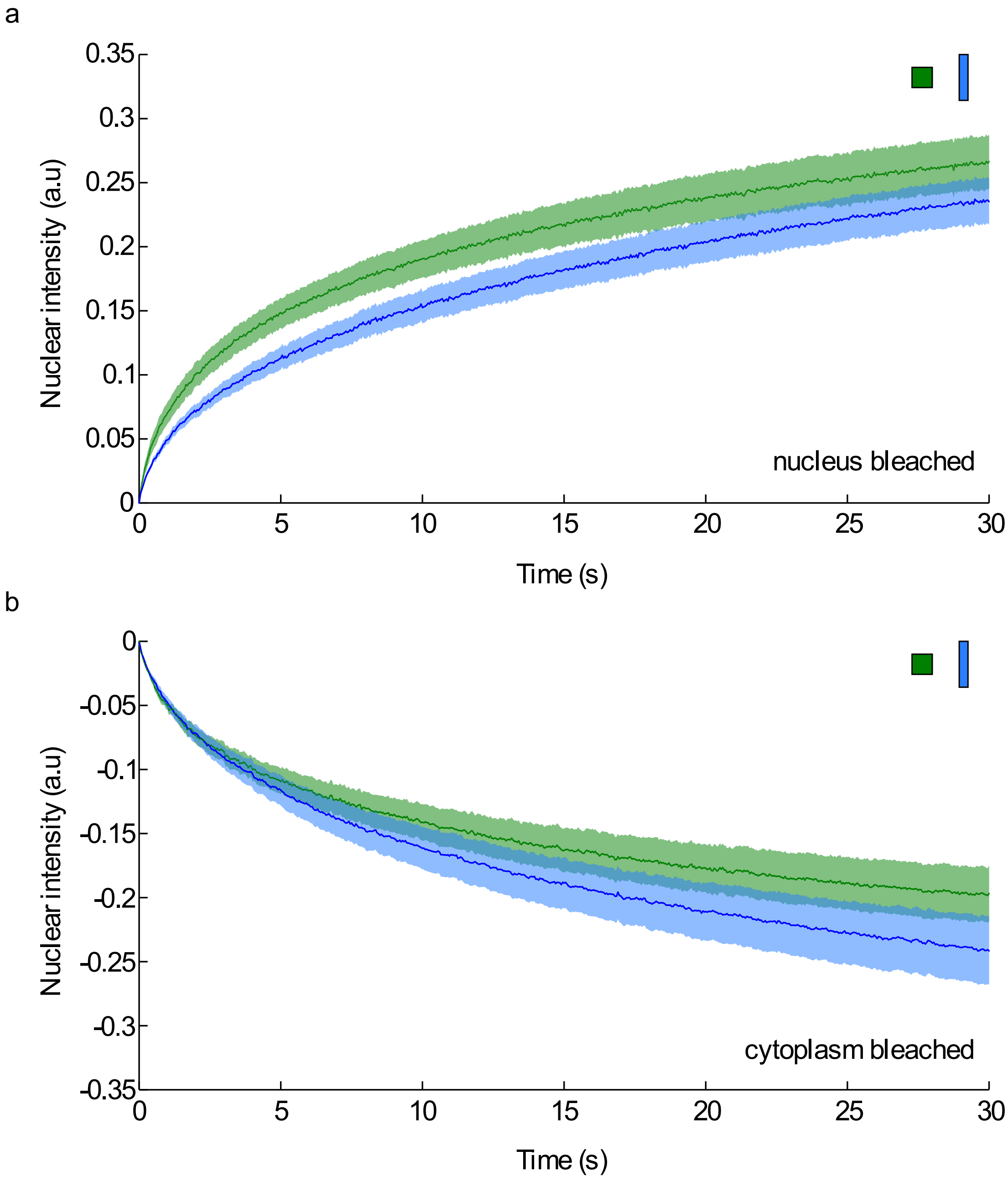

Figure S3: **Cell geometry affects the shuttling of SMYD3-eGFP.**

a) Average of recovery curves of the nuclear intensity of SMYD3-eGFP after photobleaching of the nucleus for cells spread on squares (1:1 aspect ratio, green) or rectangles (1:5 aspect ratio, blue).

b) Average of decay curves of the nuclear intensity of SMYD3-eGFP after photobleaching of the cytoplasm for cells spread on squares (1:1 aspect ratio, green) or rectangles (1:5 aspect ratio, blue).

The data are presented with +/- S.E.M (standard error mean).

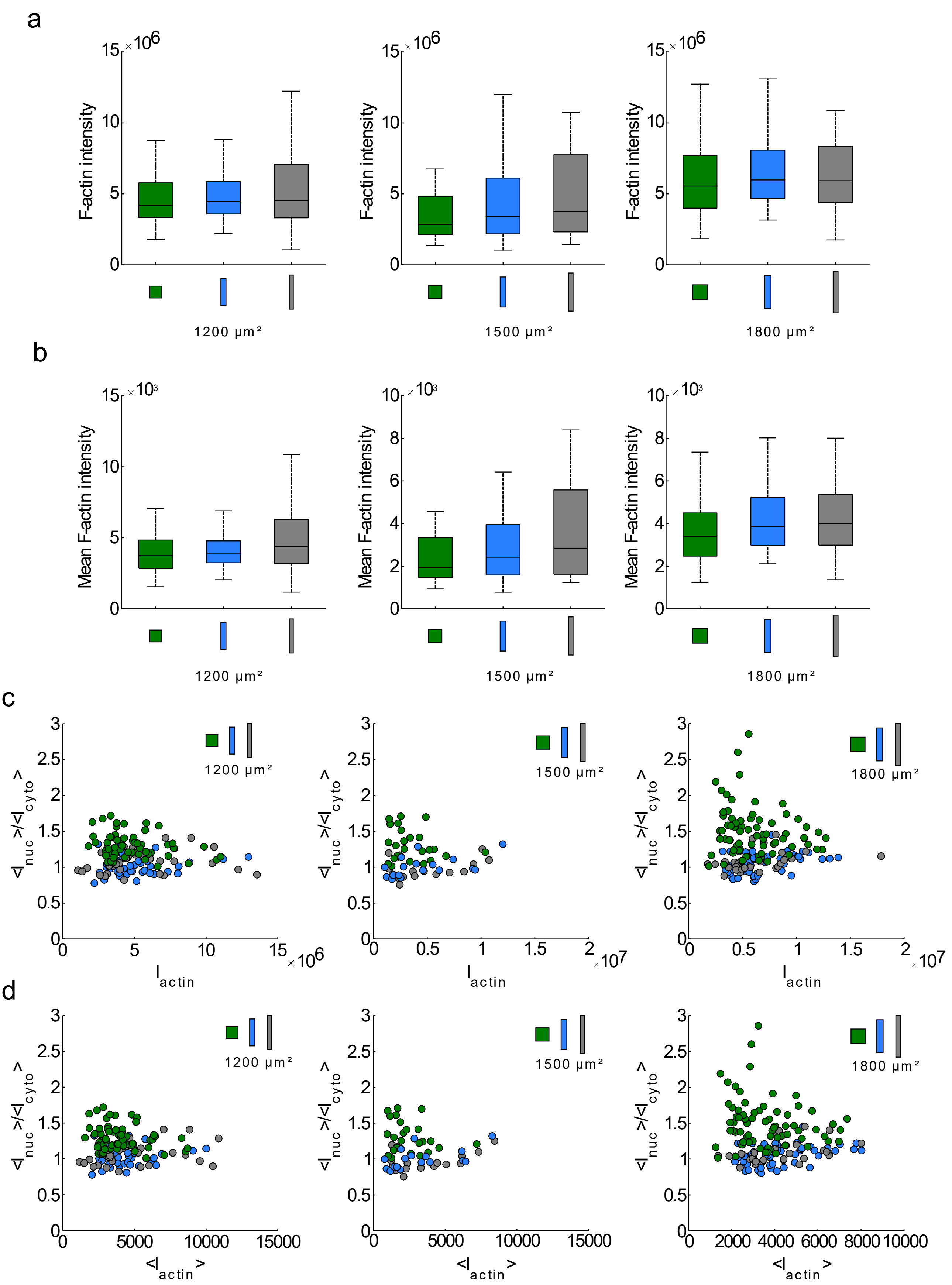
a
F-actin intensity
1200 μm²
1500 μm²
1800 μm²
b
Mean F-actin intensity
1200 μm²
1500 μm²
1800 μm²
c
$<I_{nuc}>/<I_{cyto}>$
I_{actin}
1200 μm²
1500 μm²
1800 μm²
d
$<I_{nuc}>/<I_{cyto}>$
$<I_{actin}>$
1200 μm²
1500 μm²
1800 μm²

Figure S4: **The amount of F-actin in cells spread on micropatterns is independent of cell shape or the SMYD3 distribution.**

a) Total amount of F-actin, quantified by the intensity of SiRactin staining for cells plated on micropatterns with different areas (1200 to 1800 µm²) and shapes: squares (1:1 aspect ratio, green), rectangles (1:5 aspect ratio, blue) and elongated rectangles (1:8 aspect ratio, grey).
b) Mean SiRactin intensity per pixel, under the same conditions.
c) Nuclear:cytoplasmic ($\langle I_{Nuc} \rangle / \langle I_{Cyto} \rangle$) distribution ratio for exogenous SMYD3-HA-Flag on pattern area (1500 µm²) as a function of SiRactin total intensity for each cell.
d) Nuclear:cytoplasmic ($\langle I_{Nuc} \rangle / \langle I_{Cyto} \rangle$) distribution ratio for exogenous SMYD3-HA-Flag on pattern area (1500 µm²) as a function of SiRactin mean intensity per pixel for each cell.

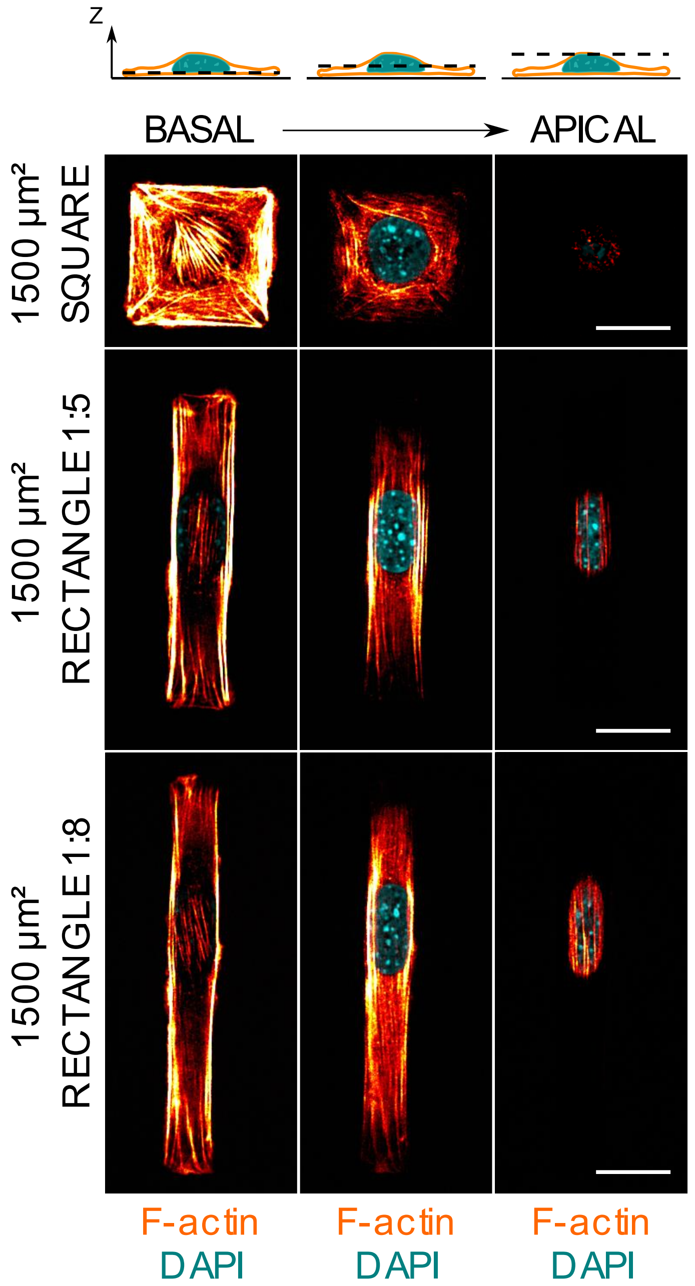

Figure S5: **Cell geometry on fibronectin micropatterns affects the organization of the actin cytoskeleton.**

Micrographs of cells spread on square (1:1 aspect ratio), rectangular (1:5 aspect ratio) and elongated rectangular (1:8 aspect ratio) micropatterns show a different organization of the F-actin networks (orangish). Scale bars: 20 µm.

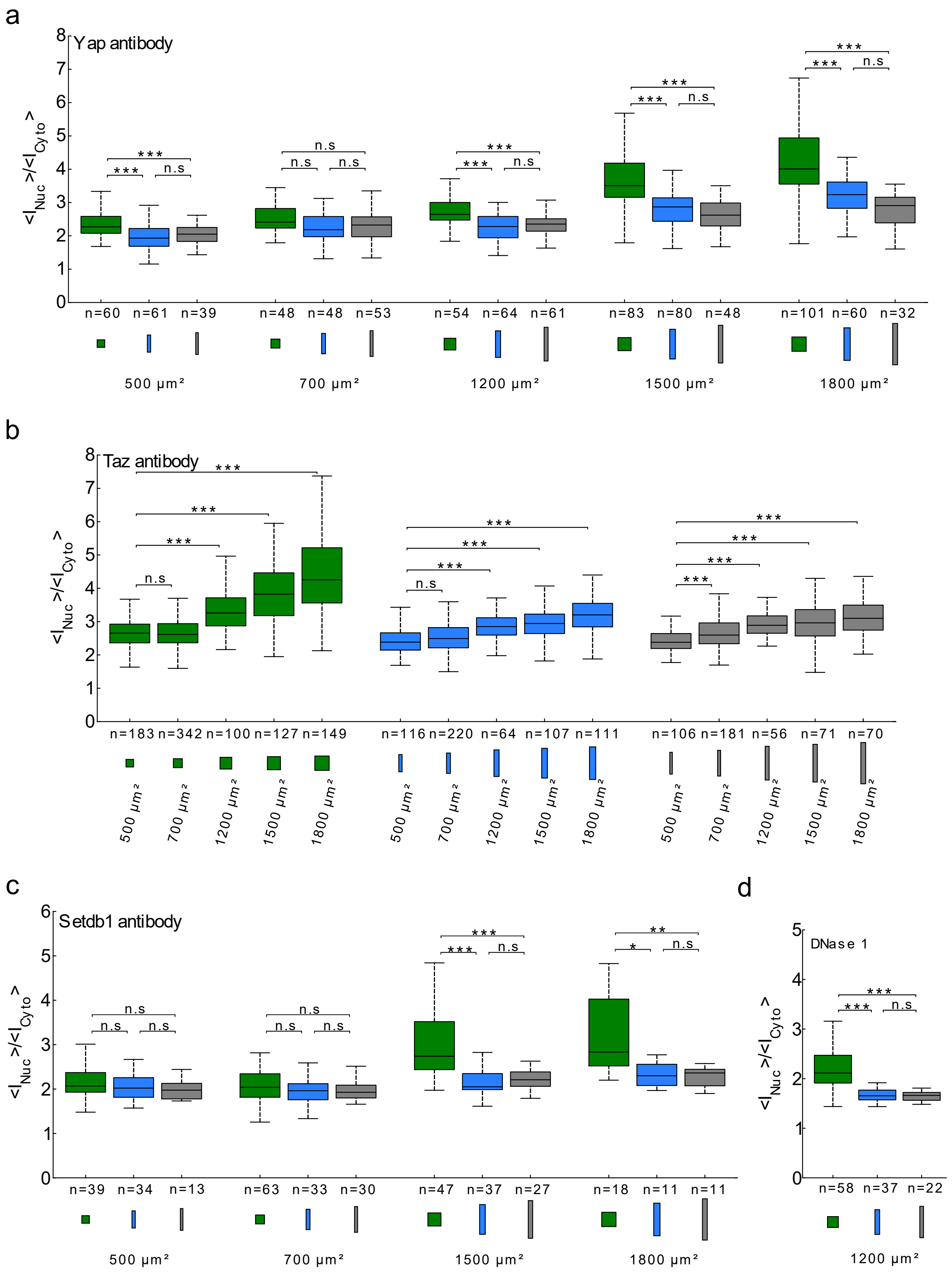
a
Yap antibody
<INuc>/<ICyto>
n=60 n=61 n=39
n=48 n=48 n=53
n=54 n=64 n=61
n=83 n=80 n=48
n=101 n=60 n=32
500 µm²
700 µm²
1200 µm²
1500 µm²
1800 µm²
b
Taz antibody
n=183 n=342 n=100 n=127 n=149
n=116 n=220 n=64 n=107 n=111
n=106 n=181 n=56 n=71 n=70
c
Setdb1 antibody
n=39 n=34 n=13
n=63 n=33 n=30
n=47 n=37 n=27
n=18 n=11 n=11
d
DNase 1
n=58 n=37 n=22
1200 µm²

Figure S6: **Cell geometry on fibronectin micropatterns regulates the localization of a range of regulatory factors.**

a) Quantification of the nuclear:cytoplasmic ($<I_{Nuc}>/<I_{Cyto}>$) distribution ratio for YAP/TAZ (anti-YAP antibody) in cells plated on different pattern areas (500-1800 µm^2) or shapes: squares (1:1 aspect ratio, red), rectangles (1:5 aspect ratio, blue) and elongated rectangles (1:8 aspect ratio, grey). The median value of the ratio is 10 to 24% higher on squares than on rectangles. An ANOVA test was performed for conditions with identical areas (500µm^2, 700µm^2, 1200µm^2). A Kruskal-Wallis test was performed for conditions with identical areas (1500µm^2, 1800µm^2).

b) Quantification of the nuclear:cytoplasmic ($<I_{Nuc}>/<I_{Cyto}>$) distribution ratio for YAP/TAZ (anti-TAZ antibody)in cells plated on different pattern areas (500-1800 µm^2) and shapes: squares (1:1 aspect ratio, red), rectangles (1:5 aspect ratio, blue) and elongated rectangles (1:8 aspect ratio, grey). A Kruskal-Wallis test was performed for conditions with identical aspect ratios.

c) Quantification of the nuclear:cytoplasmic ($<I_{Nuc}>/<I_{Cyto}>$) distribution ratio for Setdb1 protein in cells plated on different pattern areas (500-1800 µm^2) and geometries: squares (1:1 aspect ratio, red), rectangles (1:5 aspect ratio, blue) and elongated rectangles (1:8 aspect ratio, grey). For areas 1500 and 1800 µm^2, the median value of the ratio is 20-33% higher on squares than on rectangles. An ANOVA test was performed for cell spread on 500 µm^2 micropatterns. A Kruskal-Wallis test was performed for conditions with identical areas (700 µm^2, 1500 µm^2, 1800 µm^2).

d) Quantification of the nuclear:cytoplasmic ($<I_{Nuc}>/<I_{Cyto}>$) distribution ratio for DNase1 for a single pattern area 1200 µm^2 and geometries: squares (1:1 aspect ratio, green), rectangles (1:5 aspect ratio, blue) and elongated rectangles (1:8 aspect ratio, grey). The median value of the ratio is about 30% higher on squares than on rectangle patterns. A Kruskal-Wallis test was performed between conditions.

n = number of individual cells measured. * p<0.05, ** *p*<0.01, *** *p*<0.001, n.s; = not statistically significant.

Figure		number of independent repeats
1.b		5
1.d		≥6
1.e		2
2.a-d		3
2.e		1
3.b,c,e	controls	≥5
3.b,c,e	LMB	2
3.h		3
3.i		1
4.b	control	5
4.b	Latrunculin A, B	2
4.c	control	9
4.c	drugs	≥2
5.b	700 μm^2	2
5.b,d	1500, 1800 μm^2	2
S1.a		≥4
S1.b		≥5
S1.e	1200 μm^2	2
S1.e	1500, 1800 μm^2	1
S6.a,c		1
S6.b	500, 700, 1500, 1800 μm^2	2
S6.b,d	1200 μm^2	1

Table S1: Numbers of repeats of the different experiments.
